# Research Progress on Bio-Based Polyurethane-Modified Asphalt Technology

**DOI:** 10.3390/molecules31152587

**Published:** 2026-07-24

**Authors:** Yang Yang, Xiaoxue Zhang, Haiping Liu, Sitong Bie, Jie Li, Zijun Zhang, Xiaotong Qiao, Jingtao Ma

**Affiliations:** College of Civil and Architectural Engineering, Heilongjiang Institute of Technology, Harbin 150050, China; zhangxiaoxue@hljit.edu.cn (X.Z.); liuhaiping@hljit.edu.cn (H.L.); biesitong@hljit.edu.cn (S.B.); ljie1@hljit.edu.cn (J.L.); zhangzijun@hljit.edu.cn (Z.Z.); qiaoxiaotong@hljit.edu.cn (X.Q.); majingtao@hljit.edu.cn (J.M.)

**Keywords:** bio-based polyurethane, modified asphalt, pavement performance, bio-based precursor, polyol

## Abstract

Driven by the goals of carbon peaking and carbon neutrality, as well as the increasing demand for green construction materials, traditional petroleum-based asphalt can no longer fully meet the requirements of long-life and low-carbon road construction due to its strong resource dependence, susceptibility to aging, and difficulty in balancing high- and low-temperature performance. Bio-based polyurethane-modified asphalt (Bio-PUMA) uses renewable or waste biomass to construct high-performance polyurethane (PU) networks, providing a new way to improve pavement performance, reduce carbon emissions, and support the sustainable development of road materials. This paper reviews the molecular structural characteristics of bio-based precursors, including vegetable oil, rosin, and lignin, and summarizes their effects on PU network formation, asphalt microphase morphology, and pavement performance. Existing studies show that the functionality, molecular backbone, hydroxyl value, and soft-to-hard segment ratio of bio-based polyols govern the crosslinking density, phase continuity, and asphalt compatibility of polyurethane networks, thereby influencing rutting resistance, cracking resistance, aging resistance, interfacial adhesion, and mixture performance. Current challenges include unstable biomass feedstocks, difficulty in balancing low-temperature toughness and high-temperature strength, limited long-term service data, and incomplete life-cycle assessment. Future studies should focus on precursor standardization, precise molecular design, multiscale performance evaluation, and engineering validation to promote the application of Bio-PUMA in long-life, low-carbon, and large-scale road infrastructure.

## 1. Introduction

Under the goals of carbon peaking and carbon neutrality, the high-temperature production and construction processes of traditional petroleum-based asphalt pavement materials have become important aspects affecting carbon reduction in road engineering. Life-cycle assessment studies in the past three years have shown that the mixing and production stages of asphalt mixtures are key sources of greenhouse gas emissions. Among these stages, carbon emissions from asphalt mixing account for approximately 54.01% of total emissions [1]. In some maintenance projects, milling and repaving can account for approximately 87% of carbon emissions during the construction stage [2], and the production emissions of ordinary asphalt mixture plants are about 10.96 kg CO_2_e/t [3]. Therefore, the significance of bio-based polyurethane-modified asphalt lies in its potential to enhance pavement durability, reduce maintenance frequency, and improve life-cycle sustainability [4,5].

Traditional polymer modifiers, such as SBS, rubber, and epoxy resin, are widely used to improve asphalt performance; however, their dependence on non-renewable petroleum resources remains a major sustainability concern [6,7]. PU has been widely used for asphalt modification because its soft and hard segments are tunable, it exhibits high interfacial reactivity, and its mechanical properties are tailorable [8,9]. The isocyanate groups in PU can react with active hydrogen-containing components in asphalt to form a crosslinked network, thereby improving high-temperature rutting resistance, elastic recovery, and aging resistance [10,11]. In recent years, the preparation of bio-based polyurethane (BPU) from renewable resources such as vegetable oils, plant phenols, and lignin and its application in asphalt modification have become an important research direction in sustainable pavement materials [12,13]. Existing studies have shown that castor oil-based PU, lignin-based PU, and epoxidized soybean oil-based PU can improve the high- and low-temperature performance, aging resistance, and elastic recovery of asphalt to varying degrees [14,15]. These results indicate that bio-based polyurethane-modified asphalt (Bio-PUMA) has significant application potential for both performance improvement and low-carbon development.

To date, researchers worldwide have investigated polyurethane-modified asphalt, Bio-PUMA, and the road engineering applications of bio-based polyurethane (BPU) from various perspectives [16,17]. However, most of these studies focus on a single material system, specific performance attributes, or a particular engineering application aspect. A comprehensive summary is still lacking regarding the relationships among bio-based precursor structure, PU synthesis route, reactive modification mechanism, microphase morphology, and pavement performance within the Bio-PUMA system. In particular, how the functional group type, soft segment, aromatic structure, and reactivity of different biomass precursors affect PU network formation, asphalt compatibility, and service performance has yet to be fully elucidated. Furthermore, issues including dynamic covalent networks, recyclability of thermosetting PU, bio-based carbon content evaluation, life-cycle assessment (LCA), and scalable engineering applications still warrant further investigation. Against this background, this paper reviews the research progress on Bio-PUMA from two perspectives, chemical design and sustainable pavement application, covering bio-based precursors, PU synthesis routes, reactive modification mechanisms, asphalt binder and mixture performance, and functional development. This review aims to provide a reference for the rational design of Bio-PUMA and the development of sustainable pavement materials.

## 2. Chemical Basis and Precursor Diversity of Bio-PUMA

PU is a polymer containing –NHCOO– repeating units formed by the reaction between polyols and diisocyanates [8]. When used for asphalt modification, residual isocyanate (–NCO) groups in the BPU system may interact with polar functional groups in asphalt, including hydroxyl and carboxyl groups, through possible chemical reactions or intermolecular interactions. The possible reactions are shown in Equations (1) and (2). In the absence of direct spectroscopic evidence, these interactions should be regarded as a proposed mechanism rather than confirmed chemical bonding. Thus, BPU modification can be described as a physical modification process potentially assisted by chemical interactions [15].

The molecular backbone of BPU is formed through step-growth addition polymerization between polyol-derived soft segments and isocyanate-derived hard segments. The chemical structure of the polyol component directly affects the crosslinking density, the rigidity–flexibility balance, and the compatibility of the PU network with asphalt. This review summarizes three categories of polyol precursors used in Bio-PUMA.

(1) R−NCO+R′−OH→R−NHCOO−R′

(2) $$R-NCO+R\prime -COOH\to R\prime -CONH-R+CO_{2}$$

### 2.1. Vegetable Oil-Based Polyols

Vegetable oils are currently one of the most representative precursors for BPU. Their triglyceride structures contain C=C bonds, ester groups, and reactive sites that can introduce hydroxyl groups. Therefore, vegetable oil-based polyols can be prepared through several routes, such as epoxidation and ring-opening, thiol-ene click reaction, transesterification, and amidation. The hydroxyl value, functionality, molecular weight, and soft segment structure of the polyols further determine the crosslinking density, phase structure, and network continuity of PU in asphalt [18,19].

Xia et al. studied the rheological and aging properties of vegetable oil-based PU-modified asphalt and reported that the introduction of vegetable oil-based PU is an effective approach to enhance the viscoelastic behavior and aging resistance of asphalt [20]. Castor oil-based PU was used for the composite modification of bio-asphalt, and it was found that the addition of polyurethane can reduce penetration, improve low-temperature ductility, and increase both the softening point and aging resistance at a certain dosage [21]. Castor oil is mainly composed of triglycerides rich in ricinoleic acid residues, which contain hydroxyl groups in their molecular structure. These hydroxyl groups can react directly with isocyanate groups, such as those in methylene diphenyl diisocyanate, toluene diisocyanate, and isophorone diisocyanate, to form urethane linkages without prior epoxidation or ring-opening reactions. Therefore, castor oil can serve as an important bio-based polyol for preparing Bio-PUMA. The hydroxyl group reacts with the isocyanate group through an addition reaction to form a urethane linkage (-O-C(=O)-NH), as shown in Equation (3). For schematic purposes, the hydroxylated triglyceride component of castor oil is idealized as a trifunctional polyol, R(OH)_3_, although real castor oil is a mixture of triglycerides with an average hydroxyl functionality rather than a single well-defined triol. Under excess-isocyanate conditions, it can react with diisocyanates to form idealized isocyanate-terminated polyurethane prepolymers, as shown in Equations (4)–(7). Here, *R*’ represents the divalent structural residue of *IPDI*, *MDI*, or *TDI*, respectively.

(3) R−OH+R′−N=C=O→R−O−C(=O)−NH−R′

(4) $$R{\left(OH\right)}_{3}+3\;OCN-R\prime -NCO\to R{\left[-O-C(=O)-NH-R\prime -NCO\right]}_{3}$$

(5) $$3\;OCN-R_{IPDI}-NCO+\begin{array}{c} OH\\ |\\ R\\ |\\ OH \end{array}-OH\to R{\left[-O-C(=O)-NH-R\prime -NCO\right]}_{3}$$

(6) $$3\;OCN-R_{MDI}-NCO+\begin{array}{c} OH\\ |\\ R\\ |\\ OH \end{array}-OH\to R{\left[-O-C(=O)-NH-R\prime -NCO\right]}_{3}$$

(7) $$3OCN-R_{TDI}-NCO+\begin{array}{c} OH\\ |\\ R\\ |\\ OH \end{array}-OH\to R{\left[-O-C(=O)-NH-R\prime -NCO\right]}_{3}$$

During the formation of castor oil-based PU prepolymers, if chain extenders, crosslinking agents, or reactive groups such as hydroxyl and carboxyl groups in the asphalt binder are further introduced, the terminal isocyanate groups can continue to react, forming chain-extended or crosslinked structures. Therefore, castor oil-based PU can not only provide plasticizing and compatibility effects through its flexible segments in asphalt, but also build a stable polymer network via urethane bonds and subsequent crosslinking reactions.

Unlike castor oil, epoxidized soybean oil itself needs to be converted into soybean oil-based polyol through ring-opening hydroxylation before reacting with isocyanate to synthesize PU prepolymers. Shan et al. used epoxidized soybean oil as the raw material to prepare vegetable oil-based polyol (PESO) via a ring-opening reaction, and then synthesized a bio-based polyurethane prepolymer (ESPU) with IPDI [15]. As shown in Equations (8)–(10), the hydroxyl groups in PESO molecules react with the isocyanate groups in diisocyanates through an addition reaction to form urethane bonds, thereby building isocyanate-terminated PU prepolymers. In this structure, the urethane bond is represented as −O−C(=O)−NH−. If *PESO* is expressed as $PESO{\left(OH\right)}_{f}$, a polyol containing f hydroxyl groups, its prepolymerization reaction can be further expressed under excess-isocyanate conditions. Therefore, PESO reacts with $OCN-R_{IPDI}-NCO$ to form the ESPU prepolymer.

(8) PESO−OH+OCN−R′−NCO→PESO−O−C(=O)−NH−R′−NCO

(9) $$PESO{\left(OH\right)}_{f}+fOCN-R\prime -NCO\to PESO{\left[O-C(=O)-NH-R\prime -NCO\right]}_{f}$$

(10) $$PESO{\left(OH\right)}_{f}+fOCN-R_{IPDI}-NCO\to PESO{\left[O-C(=O)-NH-R_{IPDI}-NCO\right]}_{f}$$

The terminal isocyanate-type ESPU prepolymer can further react with reactive groups such as hydroxyl and carboxyl groups in the asphalt binder system, thereby improving the interfacial bonding between the PU phase and the asphalt phase. When used for asphalt modification, ESPU was found to react chemically with the reactive groups in asphalt, and a 9% dosage can significantly improve the high- and low-temperature performance, complex shear modulus, and stiffness of asphalt [15]. In addition, soybean oil-based precursors have good structural designability. Alagi et al. controlled the degree of conversion of C=C bonds in soybean oil into hydroxyl groups and adjusted the hydroxyl value of the polyol to 105 to 230 mg KOH/g with a hydroxyl functionality of 2.0~4.5 [18]. Herrán et al. used lactic acid to open the ring of epoxidized soybean oil and obtained high-functionality soybean oil-based polyols with a hydroxyl functionality of 9.0 to 12.6 [22]. Based on the above analysis, castor oil represents a precursor route with natural hydroxyl groups and direct PU formation, while epoxidized soybean oil represents a controllable route involving epoxidation, ring-opening hydroxylation, and prepolymer synthesis. Together, these two routes indicate that the key to optimizing the performance of Bio-PUMA lies in the integrated design of biomass source, hydroxyl functionality, NCO/OH ratio, ratio of soft to hard segments, and crosslinked structure, so as to achieve a balance between renewable feedstock utilization and improved pavement performance.

### 2.2. Rosin-Based Polyols

**Figure 1:**
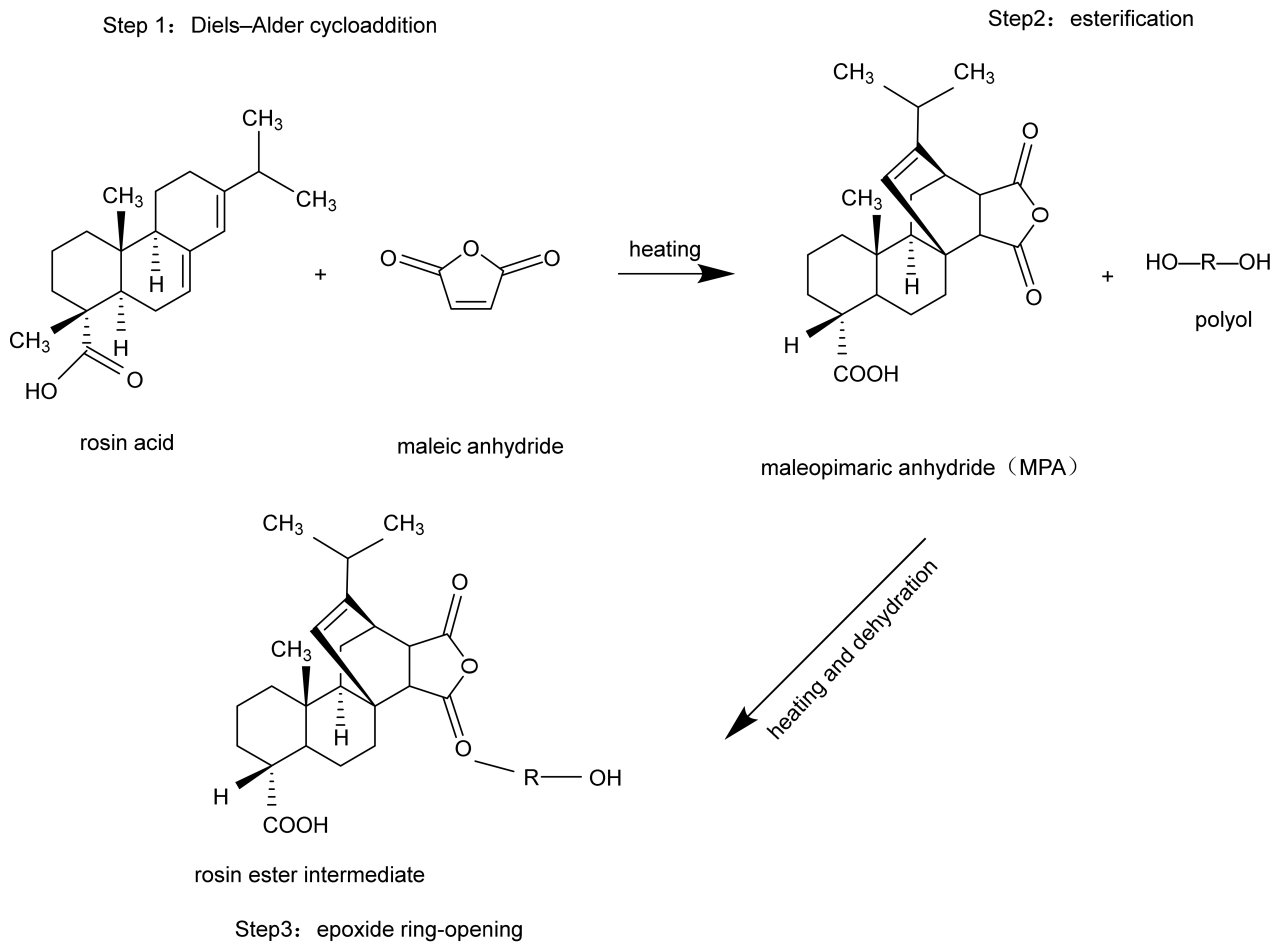
Reaction process for the synthesis of rosin-based products containing beta hydroxy ester structures.

Rosin-based polyols are representative rigid bio-based precursors in Bio-PUMA. Rosin is obtained from gum rosin, wood rosin, and tall oil rosin, and its main components are abietane-type resin acids [23]. Rosin acid undergoes a Diels–Alder cycloaddition reaction with maleic anhydride to form maleic rosin anhydride (MRA). MRA reacts with polyols via esterification to produce hydroxyl-functional rosin ester intermediates. These intermediates participate in epoxy ring-opening reactions or react with isocyanates, thereby constructing a rosin-based polyurethane crosslinked network [24]. Rosin-based products containing β-hydroxy ester structures can also be formed, and the reaction process is shown in Figure 1.

Compared with flexible plant oil-based polyols such as castor oil and soybean oil, rosin-based structures are more favorable for improving the rigidity of PU hard segments, crosslinking density, heat resistance, and hydrophobicity, thereby enhancing the high-temperature deformation resistance, water stability, and aging resistance of modified asphalt [25]. In the asphalt system, rosin-based polyols can react with the isocyanate groups of isocyanate prepolymers through an addition reaction to form a PU network containing urethane bonds. Meanwhile, their rigid hydrophobic skeleton can interact with resin and asphaltene components in asphalt, thereby mitigating the issues of poor compatibility and inadequate storage stability of bio-based components [16]. At present, research on rosin-based components in the field of road materials mainly focuses on rosin-based anhydride curing agents. Sun et al. prepared PU/epoxy resin/MRA-modified asphalt using MRA as a curing agent. The results showed that this system exhibited good mechanical properties, storage stability, and high-temperature strain recovery ability. Fourier transform infrared spectroscopy (FTIR) and atomic force microscopy (AFM) results confirmed the occurrence of the curing reaction and microstructure reconstruction [26].

### 2.3. Lignin-Based Polyols

Lignin plays three roles in Bio-PUMA: a reactive polyol precursor, a source of aromatic hard segments, and an anti-aging functional unit. Lignin is derived from lignocellulosic biomass and industrial byproducts of pulp and paper production, and it is one of the most abundant renewable aromatic resources on earth [27]. Its structure is formed by the random polymerization of phenylpropane units, such as p-coumaryl alcohol, coniferyl alcohol, and sinapyl alcohol, and contains active groups such as phenolic hydroxyl, aliphatic hydroxyl, methoxy, and carbonyl groups. Different separation processes yield technical lignins with distinct characteristics, including kraft lignin, lignosulfonate, soda lignin, and organosolv lignin, resulting in significant variations in molecular weight, hydroxyl value, sulfur content, polarity, and reactivity [28,29]. This structural heterogeneity increases the difficulty of mixture design, but also confers on lignin a tunable potential as a PU precursor. Its hydroxyl groups can partially replace petroleum-based polyether or polyester polyols to react with TDI, MDI, IPDI, or isocyanate prepolymers, while its aromatic rings can enhance hard segment rigidity, crosslinking density, and thermal oxidative stability. In addition, fractionation, hydroxypropylation, liquefaction, esterification, or blending with bio-based polyols such as castor oil and isosorbide can further adjust the solubility, hydroxyl accessibility, and NCO/OH stoichiometric balance of lignin [30].

In the asphalt system, unreacted lignin typically acts as a bio-based filler or extender component. For example, Wu et al. investigated lignin as an alternative extender for asphalt and reported that lignin incorporation increased asphalt stiffness and reduced temperature susceptibility; however, the resulting lignin-modified asphalt exhibited poor storage stability because lignin is difficult to dissolve directly in asphalt binders [31]. When incorporated into the PU reaction network, lignin can form stronger interfacial interactions with asphaltene and resin components through urethane bonds, hydrogen bonds, and aromatic structures, thereby overcoming the poor compatibility of traditional lignin-modified asphalt [32]. Peng et al. prepared lignin-based polyurethane-modified asphalt, which maintained good adhesion performance during ultraviolet aging. Its pull-off strength increased by 27.3% compared with that of neat asphalt, and FTIR analysis showed that anhydride groups and lignin aromatic rings were helpful for improving the adhesion at the asphalt–aggregate interface [33].

Overall, the three bio-based polyol precursors differ in structure and performance contribution. Vegetable oil-based polyols mainly provide flexible soft segments for low-temperature toughness, while rosin- and lignin-based polyols introduce rigid or aromatic structures that enhance high-temperature stability, aging resistance, and moisture resistance. Their structure, reaction features, and main contributions are summarized in Table 1.

## 3. Preparation Process of Bio-PUMA

### 3.1. Preparation Route

The preparation process of Bio-PUMA involves not only the PU formation reaction between bio-based polyols and isocyanates, but also the formation, diffusion, crosslinking, and phase morphology reconstruction of polyurethane chain segments in the asphalt phase [16]. Existing studies mainly employ the prepolymer method, in situ polymerization method, and reactive blending method. Compared with conventional physical blending, reactive processes can build crosslinked or semi-interpenetrating networks through urethane bonds, urea bonds, and hydrogen bonding interactions, thereby improving the dispersion, storage stability, and high-temperature deformation resistance of BPU in asphalt [34,35].

**Figure 2:**
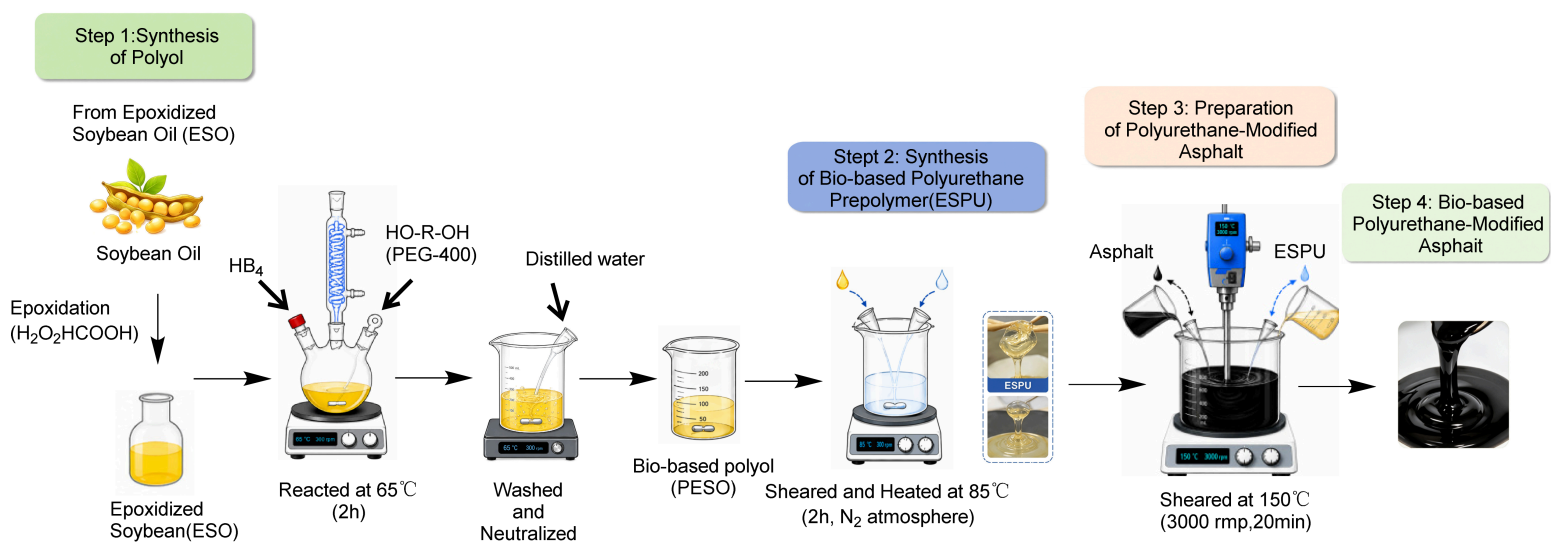
Schematic diagram of the preparation process of epoxidized soybean oil-based Bio-PUMA.

In vegetable oil-based systems, castor oil can directly participate in the PU formation reaction because it naturally contains hydroxyl groups. Gao et al. prepared Bio-PUMA via in situ polymerization, in which castor oil was first dispersed in asphalt and then MDI was added to generate BPU in situ within the asphalt phase [36]. Compared with physical blending after the prior synthesis of high-molecular-weight BPU, the in situ polymerization route is more favorable for dispersion. However, the reaction process is more dependent on temperature, feeding rate, shear mixing, and NCO/OH stoichiometric control [37]. Epoxidized soybean oil needs to be converted into vegetable oil-based polyols through ring-opening hydroxylation before the stepwise synthesis of BPU prepolymers with isocyanates [38]. As shown in Figure 2, epoxidized soybean oil is used as the raw material, and hydroxyl groups are introduced through a ring-opening reaction to prepare vegetable oil-based polyols. The polyols then react with isocyanates to form BPU prepolymers containing active isocyanate groups. After that, the prepolymer is added into heated neat asphalt, and Bio-PUMA is formed through high-speed shearing and in situ reaction [15]. Jędrzejczak et al. prepared lignin-based polyurethane-modified asphalt via reactive blending. After drying, lignin was mixed with preheated base asphalt and a catalyst to achieve preliminary dispersion in the asphalt phase [27]. Following the addition of the isocyanate prepolymer, the phenolic and aliphatic hydroxyl groups in lignin reacted with the isocyanate groups to form urethane bonds, thereby generating a lignin-based polyurethane crosslinked structure within the asphalt matrix. After curing, lignin-based polyurethane-modified asphalt was obtained [27].

In terms of process parameters, temperature, shearing time, shearing rate, stoichiometric ratio, catalyst dosage, and curing conditions jointly determine the formation rate of the PU network, phase continuity, and construction viscosity window. The study by Sun et al. on castor oil-based Bio-PUMA further showed that the BPU content controlled the transition of the system from dispersed thermoplastic modification to continuous crosslinked thermosetting modification, with about 37.5% as the initial transition threshold, and the thermosetting characteristics became more pronounced when the content exceeded 50% [39]. Therefore, the preparation process of Bio-PUMA is not merely a replacement of petroleum-based raw materials with bio-based alternatives, but also a structured design process based on the hydroxyl value of bio-based polyols, isocyanate index, ratio of soft to hard segments, reaction temperature, curing window, and asphalt compatibility. The main objective is to achieve a balance among construction workability, polymerization degree, and pavement performance enhancement.

### 3.2. Synthesis Mechanism of BPU and Bitumen

The synthesis mechanism of Bio-PUMA is essentially the result of the combined effects of PU molecular chain formation, interfacial interactions among asphalt components, and polymer network structure formation. Previous studies have shown that the performance improvement of polyurethane-modified asphalt is closely related to its soft and hard segment structure, crosslinking density, compatibility, and continuous phase structure, while the introduction of bio-based polyols further endows the system with renewable and structurally designable features [40,41]. From the perspective of preparation routes, the synthesis mechanisms of the prepolymer method, in situ polymerization method, and reactive blending method differ to some extent. In the prepolymer method, the PU main chains have been partly formed before entering asphalt. In the in situ polymerization method, PU chain segments are gradually formed inside asphalt. In the reactive blending method, PU or its precursors undergo grafting, chain extension, or crosslinking reactions with asphalt during the blending process. A summary of the synthesis mechanisms and advantages of the three methods is presented in Table 2.

## 4. Performance and Durability of Bio-PUMA

### 4.1. Effects of Modifier Characteristics on Bio-PUMA Performance

The performance of Bio-PUMA is governed by the chemical structure of the BPU modifier and its interaction with asphalt components. Relevant variables include BPU content, bio-based feedstock type, soft and hard segment structure, isocyanate index, NCO/OH ratio, crosslinking density, compatibility with base asphalt, and testing conditions. Polar groups in BPU molecules, such as –NH– and –COO–, can interact with aromatic and resin molecules in asphalt through hydrogen bonding and van der Waals interactions. These interactions enhance intermolecular forces and contribute to the stability of the internal network structure of the modified asphalt [37,38].

**Figure 3:**
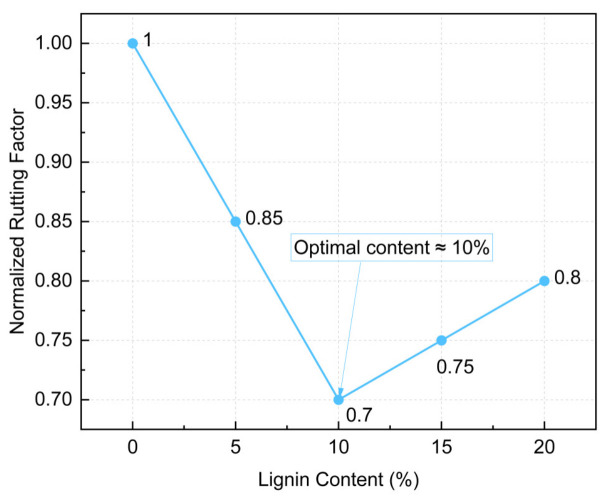
Effect of lignin content on the rutting factor of modified asphalt. Note: The data were compiled from the experimental data of Yu and Ren and the DSR test results of Wang [64,65,66].

The effect of bio-based components is not uniform across different polyurethane systems. Yu et al. and Ren et al. showed that the effect of lignin content on the rutting factor of polyurethane-modified asphalt first decreased and then increased [60]. As shown in Figure 3, the rutting factor decreased as the lignin content increased from 0% to 10% and reached its lowest value at approximately 10% lignin, indicating the weakest high-temperature rutting resistance within the tested range. When the lignin content further increased to 15–20%, the rutting factor partially recovered, suggesting some improvement in rutting resistance compared with the 10% lignin sample; however, it remained lower than that of the 0% lignin control [60]. This non-monotonic trend suggests that the high-temperature rheological response of Bio-PUMA is not linearly determined by lignin content alone.

Bio-based soft segments also affect low-temperature response. Castor oil, vegetable oil, and epoxidized soybean oil contain long aliphatic chains, which may improve the flexibility of the asphalt system. However, excessive crosslinking density can increase stiffness and reduce low-temperature ductility [15]. Therefore, the performance of Bio-PUMA is controlled by a balance between the reinforcing effect of the polyurethane network and the flexibility provided by bio-based soft segments. A higher BPU content or isocyanate index may improve high-temperature stability, but it can also reduce low-temperature deformation ability when the crosslinked structure becomes too dense [38,60,61,62,63].

### 4.2. Rutting Resistance and High-Temperature Deformation

Rutting resistance and permanent deformation are key indicators for evaluating the high-temperature performance of Bio-PUMA. The rutting factor G/sinδ is commonly used to characterize the resistance of asphalt binder to permanent deformation at high temperatures. A higher G/sinδ generally indicates a stronger resistance to high-temperature deformation under oscillatory loading.

**Figure 4:**
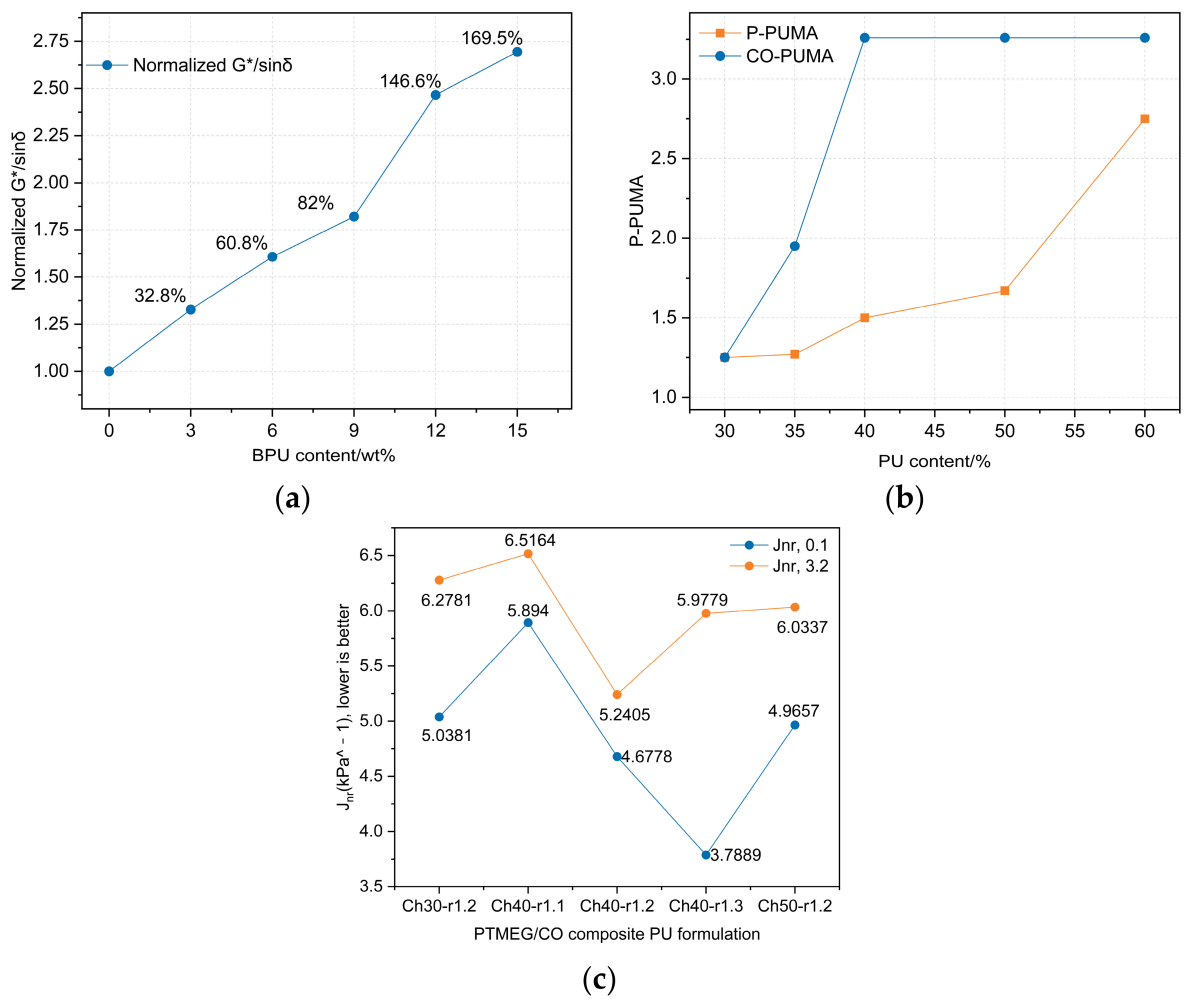
Multi-source comparison of high-temperature performance indicators of bio-based polyurethane-modified asphalt binders: (**a**) Gao et al. rutting factor at 64 °C [37]; (**b**) Sun et al. softening point [68]; (**c**) Yang et al. MSCR at 64 °C [69].

Gao et al. found that the rutting factor G*/sinδ of Bio-PUMA generally increased with BPU content [37]. As shown in Figure 4a, at 64 °C, when the BPU contents were 3 wt%, 6 wt%, 9 wt%, 12 wt%, and 15 wt%, the rutting factor increased by 32.8%, 60.8%, 82.0%, 146.6%, and 169.5%, respectively, compared with that of the base asphalt [37]. These findings indicate that vegetable oil-based polyurethane can improve the high-temperature rutting resistance of asphalt binder [67]. This improvement is mainly related to the formation of intermolecular interactions between BPU molecules and asphalt components, as well as the reinforcing effect of the polyurethane network [37,38].

Sun et al. further compared the high-temperature stability of petroleum-based polyurethane-modified asphalt and castor oil-based polyurethane-modified asphalt [68]. The softening point of castor oil-based polyurethane-modified asphalt (CO-PUMA) showed a distinct two-stage increase with increasing castor oil-based polyurethane (CO-PU) content. As shown in Figure 4b, when the CO-PU content increased from 30% to 35%, the softening point increased from 58.2 °C to 89.3 °C. When the CO-PU content reached 40% or higher, the softening point remained above 150 °C, reaching the measurable upper limit. In contrast, the softening point of petroleum-based polyurethane-modified asphalt increased only gradually from 57.5 °C to 65.6 °C within the polyurethane content range of 30% to 50% [68]. This result suggests that the trihydroxyl structure of castor oil provides more crosslinking sites, enabling CO-PU to form a continuous and dense chemically crosslinked network in asphalt, thereby suppressing high-temperature flow deformation.

The MSCR results also confirmed the high-temperature deformation resistance of CO-PUMA. CO-PUMA with CO-PU contents of 40% or higher exhibited an elastic recovery rate close to 100% and a non-recoverable creep compliance close to zero, indicating strong resistance to permanent deformation under high-temperature and heavy-load conditions [68]. Yang et al. investigated the high-temperature performance of PTMEG/CO composite polyurethane-modified asphalt from the perspective of polyurethane molecular design. As shown in Figure 4c, under the same hard segment content, PTMEG/CO composite polyurethane-modified asphalt exhibited lower non-recoverable strain than PTMEG-MDI-based polyurethane-modified asphalt, indicating that castor oil as a composite soft segment can enhance the deformation resistance of the modified asphalt [69]. Further analysis showed that when the hard segment content (ch) was 40% and the isocyanate index was 1.2, the modified asphalt exhibited a higher complex modulus and a lower phase angle, suggesting improved elastic response and high-temperature stability at this composition [69].

These results show that BPU can improve the high-temperature stability and rutting resistance of asphalt binders [37,68], but the extent of improvement depends on the type of bio-based feedstock, soft and hard segment structure, crosslinking density, base asphalt, and testing conditions [38,69]. The dosage ranges and synthesis routes reported in different studies also vary considerably, so the magnitude of improvement should not be directly compared without considering modifier content, curing conditions, and base binder properties. At the mixture level, rutting deformation is jointly governed by aggregate gradation, air void content, binder film thickness, compaction state, and loading conditions. Binder-level improvements should therefore be verified using wheel tracking, dynamic creep, repeated-load permanent deformation, Hamburg wheel tracking, or flow number tests.

### 4.3. Cracking and Fatigue Resistance

Cracking resistance of Bio-PUMA should be evaluated at both low and intermediate temperatures. Low-temperature cracking resistance is commonly characterized by low-temperature ductility, creep stiffness S, and stress relaxation ability m-value obtained from the bending beam rheometer test, as well as low-temperature tensile performance and low-temperature bending performance of asphalt mixtures [10].

**Figure 5:**
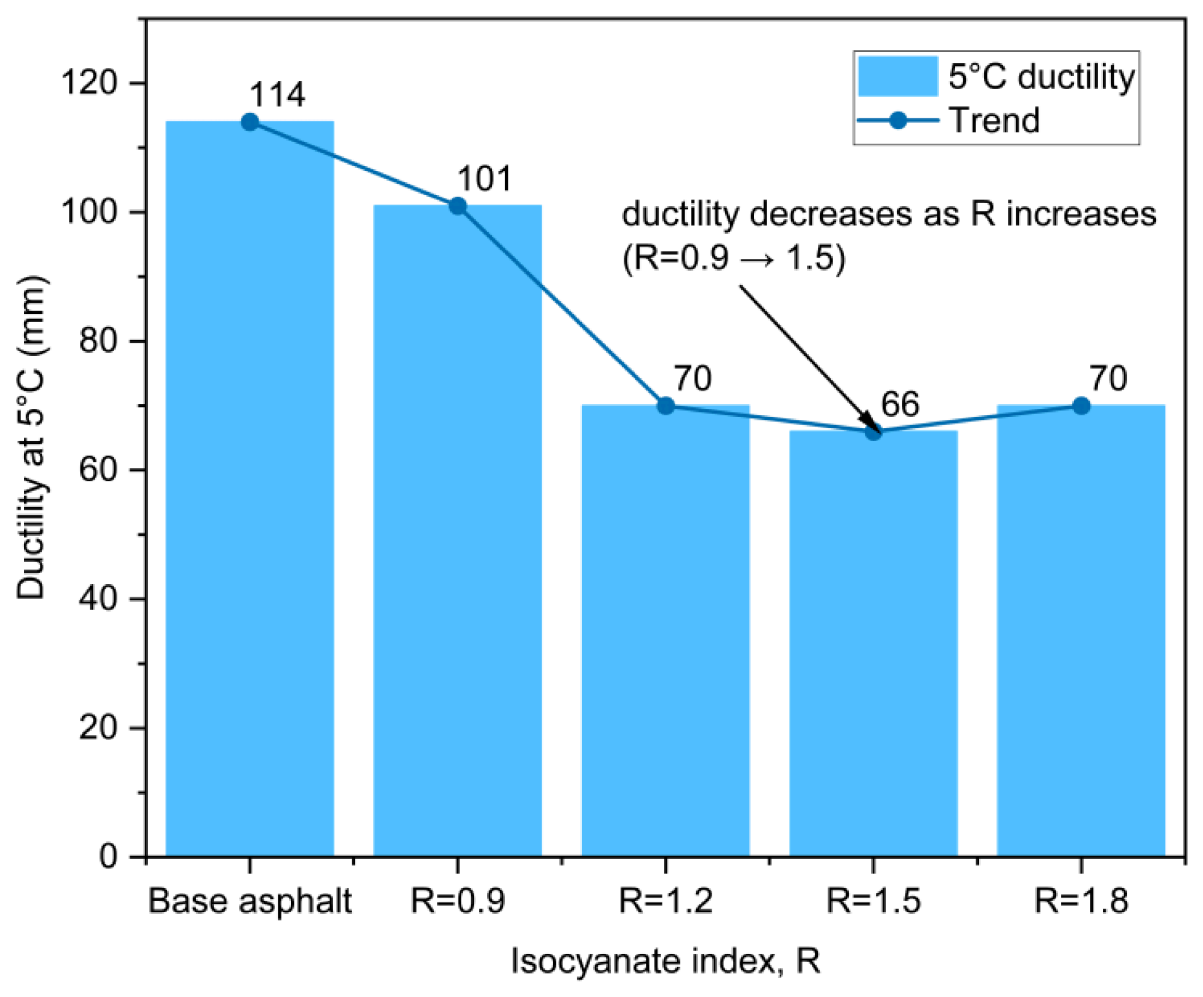
Possible decrease in low-temperature toughness with increasing isocyanate index. The figure was replotted using approximate values digitized from Figure 5b of Gao et al. [38]. The bio-based polyurethane content was 6 wt.%.

Xia et al. showed that both the high-temperature and low-temperature performance of vegetable oil-based polyurethane-modified asphalt was improved. In the aging evaluation, the 5 °C ductility of samples with different vegetable oil-based polyurethane contents varied, indicating that polyurethane content could affect the low-temperature deformation ability of modified asphalt [20]. Increasing BPU content or isocyanate index was beneficial to high-temperature performance, but it had a negative effect on low-temperature performance. Therefore, the isocyanate index was suggested not to exceed 1.5 [38]. As shown in Figure 5, when the isocyanate index was 1.5, the low-temperature toughness was the lowest, indicating that an increase in isocyanate index may reduce low-temperature ductility.

The effect of BPU on cracking resistance is closely related to the balance between soft segment flexibility and network stiffness. Bio-based soft segments such as castor oil, vegetable oil, and epoxidized soybean oil may improve low-temperature flexibility because of their long aliphatic chains. However, when the crosslinking density of PU is too high, the stiffness of the material increases and the low-temperature ductility may decrease [15]. Therefore, the low-temperature performance of Bio-PUMA does not increase monotonically with modifier content. It is jointly controlled by soft segment content, hard segment content, isocyanate index, crosslinking density, and compatibility [62,63].

At the mixture level, BPU modification may affect cracking and fatigue behavior through changes in binder stiffness, aggregate–binder adhesion, and viscoelastic response. Han et al. studied the dynamic modulus, static modulus, and creep performance of bio-based polyurethane-modified asphalt mixture. The results showed that the mixture could maintain good elastic characteristics over a wide temperature range and improve mechanical stability [70]. Therefore, future studies should focus on the relationships among bio-based soft segment structure, isocyanate index, crosslinking density, and low-temperature cracking indicators. In addition, the bending beam rheometer test, low-temperature ductility test, low-temperature bending test, and micromorphology analysis should be integrated to establish an evaluation system for the low-temperature performance of bio-based polyurethane-modified asphalt [71,72,73,74].

### 4.4. Moisture Damage, Aging Resistance and Environmental Durability

Environmental durability of Bio-PUMA involves moisture damage, asphalt–aggregate adhesion, oxidative aging, ultraviolet aging, and hydrothermal stability. Moisture damage is especially important at the mixture scale because stripping and interfacial debonding are affected not only by binder chemistry, but also by aggregate mineralogy, air void content, binder film thickness, compaction state, and freeze–thaw conditioning.

Kazemi et al. synthesized bio-based polyurethane using castor oil, TDI, and diethylene glycol. The splitting tensile strength ratios of the mixtures increased by 19%, 40%, and 49% at dosages of 3%, 6%, and 9%, respectively. The bonding strengths of the 9% dosage system under dry and wet conditions increased by 55.73% and 37.93%, respectively, indicating that BPU can enhance asphalt–aggregate interface adhesion and improve resistance to water damage [60].

The aging resistance of Bio-PUMA is generally associated with the restriction of light component migration and oxidation by the PU network, as well as the antioxidant structures of some bio-based precursors. Lignin, quercetin, cashew nut shell liquid, and other components contain aromatic rings, phenolic hydroxyl groups, or unsaturated side chains, which can contribute to free radical scavenging, ultraviolet absorption, and polar interactions, thereby delaying thermal oxidative aging and ultraviolet aging [14]. After comparing bio-based components such as chitosan, cashew nut shell liquid, lignin, and quercetin, Cao et al. found that all types of bio-based polyurethane systems could improve the fatigue toughness and aging resistance of modified asphalt. Among them, lignin-based polyurethane-modified asphalt showed the best performance, and its rutting factor aging index and sulfoxide index growth rate were 78% and 92% lower than those of conventional PU-modified asphalt, respectively [14].

Although these results indicate that BPU can improve aging resistance and moisture durability, the available evidence is still concentrated on short-term laboratory indicators. Long-term hydrothermal stability, ultraviolet–thermal coupling, moisture–aging interaction, and performance retention after repeated environmental conditioning remain insufficiently clarified. Possible biological or microbial deterioration of bio-based asphalt materials may also deserve attention, but current evidence is not sufficient to treat mold resistance as a major performance category for Bio-PUMA.

### 4.5. Self-Healing and Integrated Pavement Performance

Self-healing is an important functional property of Bio-PUMA. The self-healing of asphalt materials usually involves crack interface wetting, molecular diffusion, structural rearrangement, and recovery of bonding strength. It is affected by material composition, temperature, damage degree, healing time, loading history, and aging state [75]. For polyurethane-modified asphalt, dynamic covalent bonds and reversible non-covalent interactions can improve damage repair through bond exchange, network reconstruction, and recovery of interfacial bonding [76].

Cao et al. prepared and characterized polyurethane-modified asphalt containing dynamic covalent bonds, showing that the dynamic bond structure can endow the polyurethane network with a certain reconfigurability and improve the damage repair ability and service stability of modified asphalt [77]. The castor oil-based polyurethane prepared by Wei et al. introduced dynamic reversible structures such as disulfide bonds, Diels–Alder bonds, transesterification bonds, and imine bonds into the bio-based polyurethane network (vitrimer)-modified asphalt. The strength recovery ratio after the first self-healing cycle exceeded 75% at 80 °C and 0.3 MPa [78]. The studies by Ban et al. and Shu et al. also showed that dynamic exchange of disulfide bonds could significantly improve the bonding recovery and fatigue healing ability of polyurethane-modified asphalt [79,80].

Self-healing should not be interpreted as an isolated advantage. A network with sufficient mobility may favor healing, but excessive mobility may reduce high-temperature stability. Healing efficiency also depends on the rest period, temperature, damage level, aging condition, and moisture exposure. Therefore, the evaluation of self-healing should be connected with rutting resistance, cracking resistance, aging resistance, and moisture durability rather than being reported as a single recovery index.

Research on Bio-PUMA has gradually extended from the binder scale to the mixture level. The evaluation indices include dynamic modulus, static modulus, creep, water stability, adhesion performance, and resistance to permanent deformation. The BPA-16 mixture designed by Han et al. showed dynamic moduli that were 8.7% and 30.4% higher than those of conventional PU-modified mixture and base asphalt mixture, respectively, at 25 Hz and −20 °C. No failure stage was observed during the 3600 s creep process, and the creep strain was the lowest, indicating that lignin- and chitosan-based BPU can help improve the low-temperature mechanical stability and resistance to permanent deformation of mixtures [70].

Existing results show that Bio-PUMA has potential in high-temperature stability, interface adhesion, aging resistance, and functional self-healing. However, low-temperature toughness, fatigue durability, construction viscosity, storage stability, long-term hydrothermal stability, and engineering-scale adaptability remain key constraints. Future studies should move beyond single-index performance improvement and evaluate Bio-PUMA through a balanced framework covering rutting resistance, cracking and fatigue resistance, moisture durability, aging resistance, healing capacity, workability, and mixture-scale pavement performance.

## 5. Sustainability and Recycling of Bio-PUMA

A key limitation arises from the crosslinked structure of polyurethane-modified asphalt. Crosslinking can improve elasticity, cohesion, rutting resistance, and aging resistance, which are desirable for pavement performance. However, highly crosslinked thermoset networks cannot melt and flow like thermoplastic modifiers such as SBS. Once the polyurethane network is formed, molecular mobility is restricted, and conventional hot recycling or reblending may become difficult. Consequently, the network structure that contributes to improved mechanical performance may also limit reprocessability and create additional barriers to the reuse of reclaimed asphalt pavement containing Bio-PUMA. The end-of-life issue is particularly important for thermosetting polyurethane systems. Miravalle et al. noted that thermoset PU recycling has traditionally relied on destructive approaches such as glycolysis, acidolysis, thermal decomposition, combustion, or landfilling, all of which may involve material degradation, additional emissions, or loss of material value [81]. For Bio-PUMA, the use of bio-based monomers does not automatically resolve this problem. If the modified asphalt cannot be effectively recycled or reused, its sustainability claim may be weakened despite its renewable origin.

Dynamic covalent networks offer a possible route to address this conflict. Covalent adaptable networks can maintain crosslinked structures under service conditions while allowing bond exchange and network rearrangement under appropriate stimuli. Miravalle et al. demonstrated that a commercially available bio-based thermoset polyurethane could be reworked up to three times while largely maintaining its crosslinked structure and thermal properties [81]. In asphalt systems, Lyu et al. showed that disulfide-crosslinked poly(urea-urethane) elastomer could improve the self-healing capacity of asphalt binder through dynamic disulfide exchange, while also enhancing rutting and cracking resistance at suitable dosages [82]. These findings suggest that dynamic urethane, disulfide, imine, ester-exchange, or Diels–Alder chemistries may be incorporated into future Bio-PUMA designs to improve reprocessability and damage tolerance.

However, self-healing should not be equated with recyclability. The recyclability of Bio-PUMA still requires direct validation after aging, repeated heating, reclaimed asphalt incorporation, and mixture-level performance testing. Future studies should therefore evaluate modulus retention, phase stability, crosslink density evolution, fatigue recovery, rutting resistance, and emissions after recycling. At the current stage, Bio-PUMA should be presented as a potentially sustainable material, not an inherently recyclable one.

## 6. Conclusions and Prospects

Bio-PUMA combines renewable biomass precursors with the reactive modification mechanism of polyurethane, providing a highly promising pathway for the low-carbon replacement and high-performance development of traditional petroleum-based polymer-modified asphalt. Based on the systematic review of the existing literature, the following main conclusions can be drawn:

(1) The structure of bio-based precursors is a key factor affecting the performance of Bio-PUMA. Biomass raw materials, such as vegetable oils, lignin, rosin, chitosan, and cashew nut shell liquid, have different hydroxyl values, functionalities, molecular backbones, and polar groups, which lead to different effects on the soft and hard segment ratio of polyurethane, crosslinking density, and compatibility with asphalt. Among them, vegetable oil-based polyols are helpful for improving flexibility and low-temperature performance, while rigid precursors such as lignin and rosin are more effective in improving high-temperature stability, aging resistance, and water stability.
(2) The performance improvement of Bio-PUMA comes from the combined effect of chemical reactions and microphase structure reconstruction. The isocyanate groups in bio-based polyurethane can react with active groups in asphalt, such as hydroxyl and carboxyl groups, and enhance the interfacial bonding between the polyurethane phase and asphalt phase through urethane bonds, hydrogen bonds, and crosslinked networks. The prepolymer method, in situ polymerization method, and reactive blending method can all achieve effective modification, but the NCO/OH ratio, reaction temperature, shear conditions, and curing conditions should be properly controlled to ensure system stability and construction adaptability.
(3) Bio-PUMA can significantly improve the overall pavement performance of asphalt, but the balance among different properties still needs to be considered. Existing studies show that Bio-PUMA has clear advantages in improving high-temperature rutting resistance, elastic recovery, aging resistance, interface adhesion, and water stability of mixtures. At the same time, the proper introduction of flexible bio-based segments also helps to improve low-temperature toughness. However, excessively high crosslinking density may reduce low-temperature ductility or increase construction viscosity. Therefore, its optimal design should focus on coordinating the relationships among high-temperature strength, low-temperature cracking resistance, construction flowability, and long-term durability.

Although Bio-PUMA shows good application potential, there are still some problems that need to be solved. The sources of biomass raw materials are complex, and different batches may show large differences in hydroxyl value, functionality, and reactivity, which can easily lead to insufficient stability of the polyurethane network structure and its properties. There is still a certain conflict between high-temperature stability and low-temperature toughness. Although high crosslinking density is helpful for improving strength and durability, it can also limit the mobility of molecular chains, thereby affecting low-temperature cracking resistance. Most existing studies are still focused on the binder scale and laboratory testing stage, while research on hydrothermal coupled aging, fatigue damage evolution, and engineering adaptability under long-term service conditions is still relatively limited. In addition, although the highly crosslinked thermosetting network of Bio-PUMA enhances mechanical performance and durability, it also makes recycling and end-of-life management more difficult than for thermoplastic modifiers such as SBS. Current research remains largely focused on service performance, while recyclability, reprocessing routes, disposal strategies, life-cycle carbon benefits, and bio-carbon content evaluation are still insufficiently investigated.

Future research on Bio-PUMA should further strengthen the standardization of bio-based precursors and the precise design of molecular structures, with a focus on the coordinated control of hydroxyl value, NCO/OH stoichiometry, soft and hard segment ratio, and crosslinked structure, so as to achieve a balance among high-temperature strength, low-temperature toughness, and construction performance. Improving the recyclability of highly crosslinked Bio-PUMA systems also deserves particular attention. Dynamic covalent network technologies, including covalent adaptable networks (CANs) and vitrimer-like polyurethane systems, may enable bond exchange and network rearrangement, allowing reprocessing and recycling while retaining the mechanical benefits of crosslinked structures. At the same time, life-cycle assessment (LCA), carbon emission accounting, and field demonstration projects should be combined to systematically evaluate the long-term environmental and economic benefits of Bio-PUMA in green and low-carbon road construction, thereby promoting its development toward long service life, low-carbon design, and large-scale engineering application.

## Figures and Tables

**Table 1:** Concise comparison of three bio-based polyol precursors for Bio-PUMA.

Precursor Type	Vegetable Oil-Based Polyols: Castor Oil, Epoxidized Soybean Oil	Rosin-Based Polyols	Lignin-Based Polyols
Structure	Long aliphatic chains and ester groups	Rigid hydrophobic abietane-type skeleton	Aromatic macromolecular structure containing phenolic and aliphatic hydroxyl groups
Reaction features	Castor oil contains natural hydroxyl groups, while epoxidized soybean oil usually requires ring-opening hydroxylation before PU synthesis	Commonly modified through Diels–Alder reaction, esterification, or epoxy ring-opening to introduce reactive hydroxyl groups	React with isocyanates or isocyanate prepolymers after modification or blending
Main contribution	Mainly provide flexible soft segments, improving flexibility, low-temperature toughness, viscoelasticity, and aging resistance; excessive crosslinking may increase stiffness and reduce low-temperature ductility	Introduce rigid segments and improve crosslinking density, high-temperature stability, water resistance, and aging resistance; excessive rigidity may weaken low-temperature cracking resistance	Act as a reactive polyol, aromatic hard segment, and anti-aging unit, improving stiffness, adhesion, thermal/UV aging resistance, and water stability; structural heterogeneity and poor solubility may affect compatibility and storage stability

**Table 2:** Comparison of the prepolymer method, in situ polymerization method, and reactive blending method [42,43,44,45,46,47,48,49,50,51,52,53,54,55,56,57,58,59].

Method	Defining Feature	Reaction/Process Feature	Potential Advantages	Main Risks/Required Validation
Prepolymer method	PU prepolymer is synthesized before asphalt blending.	NCO-terminated or partially reactive PU prepolymer is blended with asphalt; residual NCO may react with accessible polar groups or form physical/chemical associations [43,45].	High controllability of PU architecture; suitable for structure–property studies; potential improvement in high-temperature rheology and storage stability [50,51].	Additional synthesis and storage step; moisture sensitivity; premature curing and viscosity growth; possible low-temperature embrittlement. Residual NCO, molecular weight, storage stability, morphology, and rheological performance should be verified [58,59].
In situ polymerization method	PU chains form mainly inside the asphalt matrix.	Isocyanate, polyol, chain extender, and catalyst react during asphalt mixing; network formation depends on the balance between reaction rate and dispersion [42,43,44].	Avoids prepolymer storage; may improve initial dispersion and interfacial coupling; potential enhancement of rutting resistance and elastic recovery [42].	Local stoichiometry is difficult to control; rapid reaction may cause microgels, phase separation, or uncontrolled viscosity growth [44]. NCO conversion, curing rheology, network formation, morphology, and low-temperature performance should be verified [56,57].
Reactive blending method	A low-viscosity reactive modifier is blended directly with asphalt.	Reactive groups may couple with accessible asphalt polar fractions or induce stronger physical association; complete PU network formation is not necessarily required [52,53].	Simple processing; lower viscosity; potentially reduced mixing temperature and energy; improved high-temperature performance at suitable dosage [46,47,48,49].	Strong dependence on asphalt composition; reaction extent is uncertain; excessive reactivity may cause over-crosslinking, asphaltene aggregation, poor workability, or low-temperature deterioration [54]. Reactive controls and spectroscopy, morphology, storage, and rheological tests are required [55].

## Data Availability

No new data were created or analyzed in this study. Data sharing is not applicable to this article.

## Abbreviations

The following abbreviations are used in this manuscript:

Bio-PUMA	Bio-based polyurethane-modified asphalt
PU	Polyurethane
BPU	Bio-based polyurethane
MDI	Methylene diphenyl diisocyanate
TDI	Toluene diisocyanate
IPDI	Isophorone diisocyanate
PESO	Prepared vegetable oil polyol
ESPU	Bio-based polyurethane prepolymer
MRA	Maleic rosin anhydride
FTIR	Fourier transform infrared spectroscopy
AFM	Atomic force microscopy
